# Mechanical, Swelling, and Structural Properties of Mechanically Tough Clay-Sodium Polyacrylate Blend Hydrogels

**DOI:** 10.3390/gels3010010

**Published:** 2017-02-25

**Authors:** Hiroyuki Takeno, Yuri Kimura, Wataru Nakamura

**Affiliations:** Department of Chemistry and Chemical Biology, School of Science and Technology, Gunma University, Kiryu, Gunma 376-8515, Japan; t11301062@gunma-u.ac.jp (Y.K.); t10801189@gunma-u.ac.jp (W.N.)

**Keywords:** clay, blend hydrogels, toughness, swelling properties, synchrotron small-angle X-ray scattering

## Abstract

We investigated the mechanical, swelling, and structural properties of mechanically tough clay/sodium polyacrylate (PAAS) hydrogels prepared by simple mixing. The gels had large swelling ratios, reflecting the characteristics of the constituent polymer. The swelling ratios initially increased with the increase of the swelling time, and then attained maximum values. Afterwards, they decreased with an increase of the swelling time and finally became constant. An increase in the clay concentration lead to a decrease in the swelling ratios, whereas an increase in the PAAS concentration lead to an increase in the swelling ratios. Tensile measurements indicated that the toughness for clay/PAAS (*M*_w_ = 3.50 × 10^6^) gels was several hundred times larger than that of clay/PAAS (*M*_w_ = 5.07 × 10^5^) gels, i.e., the use of ultra-high molecular weight PAAS is essential for fabricating mechanically tough clay/PAAS blend hydrogels. Synchrotron small-angle X-ray scattering (SAXS) results showed that the SAXS intensity measured at small scattering angles decreased with an increase in the clay concentration, indicating that the interparticle interactions were more repulsive at higher concentrations. The decrease of the scattering intensity at high clay concentrations was larger for the clay/PAAS (*M*_w_ = 5.07 × 10^5^) gel system than for the clay/PAAS (*M*_w_ = 3.50 × 10^6^) gel system.

## 1. Introduction

There has been an increased interest in the material properties of mechanically tough hydrogels. Nanocomposite hydrogels composed of clay and a polymer are well-known as hydrogels with excellent mechanical properties. The nanocomposite hydrogels have potential applications in fields such as biomedical engineering and electronic devices [1]. Nanocomposite hydrogels are prepared by free-radical polymerization of alkylacrylamide in the presence of clay particles, e.g., they are extraordinarily stretchable (~1500%) and mechanically tough [2,3,4]. In recent years, we have found that blend hydrogels composed of clay and sodium polyacrylate (PAAS) prepared by simple mixing did not fracture even after 90% compression tests or twisting of the gels [5,6]. For preparation of the clay/PAAS blend hydrogels, a dispersant such as tetrasodium pyrophosphate (TSPP) was used to prevent the formation of aggregates of clay particles, e.g., a house of card structure [7]. Moreover, the effects of the polymer molecular weight and composition on the mechanical properties of the blend hydrogels has been investigated [6,8]. From these studies, it has been concluded that it is necessary to use polymers with molecular weights higher than a few million grams per mole and to disperse clay particles for the production of mechanically tough clay/PAAS blend hydrogels. In addition, the previous studies have suggested that polyacrylate anions adsorb on the edge of the clay particles [5,8]. Thus, though the mechanical properties of the blend hydrogels and key factors used to achieve toughness have been elucidated to some degree, studies on other properties such as swelling behavior, quantification of toughness, and the effect of clay concentration on structures of the gels remain poorly understood. In particular, as PAAS is well-known as a super absorbent polymer, the swelling properties of the clay/PAAS blend hydrogels are also expected to be important.

In this study, in order to clarify the previously stated issues, we investigated the mechanical, swelling, and structural properties of the clay/PAAS blend hydrogels using tensile measurements, time-course measurements of swelling ratios, and synchrotron small-angle X-ray scattering (SAXS) measurements.

## 2. Results and Discussion

**Figure 1:**
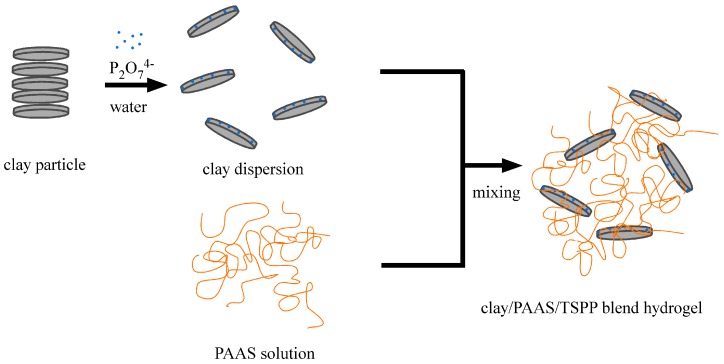
A schematic representation for the preparation of clay/PAAS blend hydrogels.

We show a schematic representation for the preparation of clay/PAAS blend hydrogels in Figure 1. The preparation method is very simple. First, clay particles are mixed in a dispersant (TSPP) aqueous solution. As the clay particles have negatively charged surfaces and positively charged edges, it is expected that pyrophosphate anions adsorb on the positively charged edges of the clay particles. As a consequence, the clay particles are dispersed in water. The clay dispersion is added to a PAAS aqueous solution little by little, and is mixed with the PAAS solution by stirring the mixture. PAAS is also expected to adsorb on the positively charged edges of the clay particles, because it is an anionic polyelectrolyte. Thus, the clay/PAAS blend hydrogels are prepared by simple mixing.

**Figure 2:**
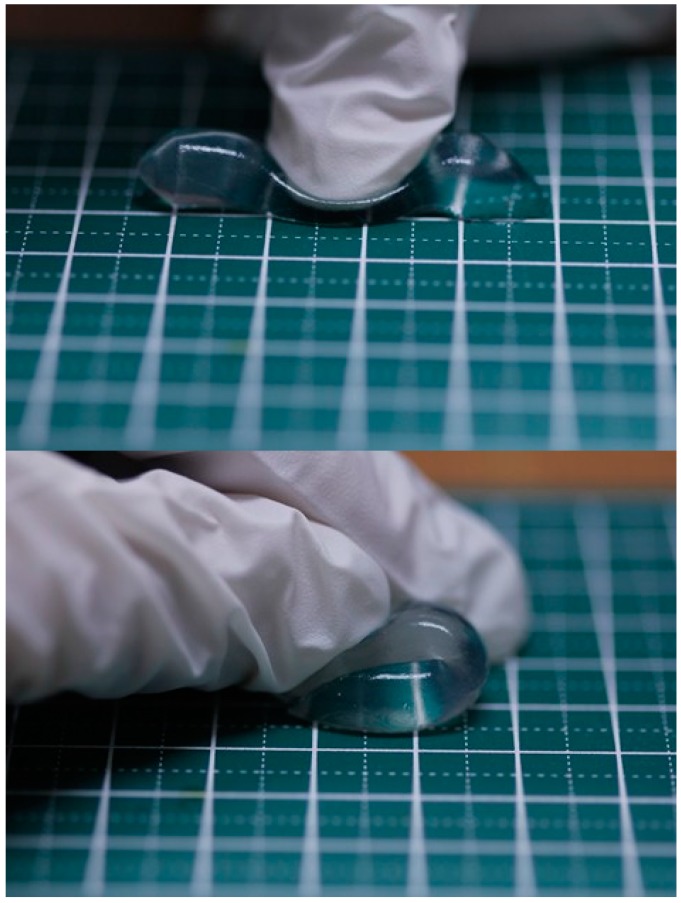
Pictures of a 5 wt % clay/1 wt % PAAS3500K/0.5 wt % TSPP blend hydrogel. A pressed gel (**top**) and a bent gel (**bottom**).

Figure 2 depicts pictures of a 5 wt % clay/1 wt % PAAS3500K/0.5 wt % TSPP blend hydrogel. These pictures show that the gel does not fracture, even if it is strongly pressed or is bent. Thus, the blend hydrogel has mechanical toughness.

### 2.1. Time Course of Swelling Ratios for Clay/PAAS Hydrogels

The swelling behavior of ionic hydrogels is affected by the following components; the free energy change of mixing, the free energy change due to the network elasticity, and the free energy change resulting from the presence of mobile ions [9,10,11].

**Figure 3:**
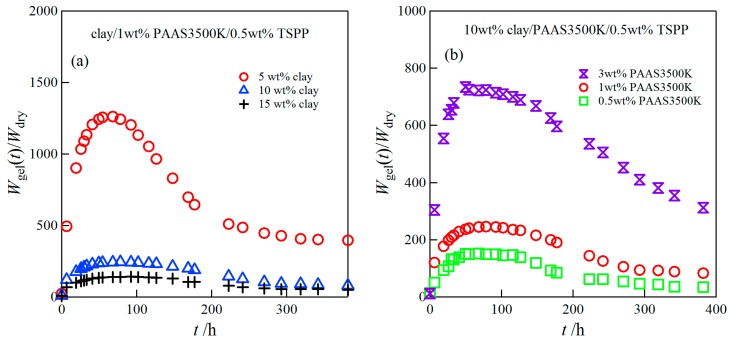
Time course of the swelling ratios for clay/PAAS3500K hydrogels at various clay concentrations (**a**) and at various PAAS concentrations (**b**).

Figure 3 depicts the time course of swelling ratios *W*_gel_(*t*)/*W*_dry_ for clay/1 wt % PAAS3500K hydrogels at various clay concentrations (a) and for 10 wt % clay/PAAS3500K hydrogels at various PAAS concentrations (b) in water. The swelling ratios of the clay/PAAS hydrogels initially increased with time, and then reached a maximum value, e.g., they attained their maximum values at 68–80 h in the case of the 5–15 wt % clay/1 wt % PAAS3500K hydrogels. The swelling kinetics of a gel is described by a collective diffusion equation with a diffusion coefficient, which is defined as the ratio of the longitudinal osmotic modulus of the network to the frictional coefficient [12]. Therefore, in general the swelling dynamics of the gel is slow. In the case of the clay/polymer blend hydrogel, a rearrangement of clay particles may occur during the course of the swelling, because the clay particles are linked to the polymers by physical ionic interactions. The maximum ratio exceeded 1000 for the 5 wt % clay/1 wt % PAAS3500K hydrogels. Afterwards, they decreased with time, i.e., deswelling took place, and finally became constant. Such swelling-deswelling behavior has also been reported by Haraguchi et al. for nanocomposite hydrogels prepared by free-radical polymerization of acrylamide, *N*,*N*-dimethylacrylamide, and *N*-isopropylacrylamide, when the clay concentration is above a critical concentration [13,14]. They showed that the deswelling behavior occurred due to the diffusion of mobile sodium ions from the inside of the network into the outer water [14]. The mobile sodium ions were replaced by hydrogen ions. The mechanism should also occur for clay/PAAS blend hydrogels. The replaced hydrogen ions may associate with the negative ions on the clay surface and the carboxylate anions of PAAS, so that the mobile ions in the network decrease and therefore the swelling ability of the hydrogel is reduced. Thus, the swelling-deswelling behavior for clay/PAAS blend hydrogels is similar to that observed in the nanocomposite clay/poly(alkylacrylamide) gel, although the former swelling ratio is much greater than that of the latter gel, reflecting a super-absorbent character of PAAS. As seen in Figure 3a, both the maximum values of the swelling ratios and the constant values at long swelling times decreased with the increase of the clay concentrations. The increase of the clay concentration causes the increase of the cross-link densities as has been reported in a previous study [5], and consequently leads to the decrease of the swelling ratios. On the other hand, as expected, the swelling ratios increased with the increase of the anionic polymer concentrations due to the increase in the ionic contribution to the osmotic pressure.

### 2.2. Mechanical Properties of Clay/PAAS Hydrogels

**Figure 4:**
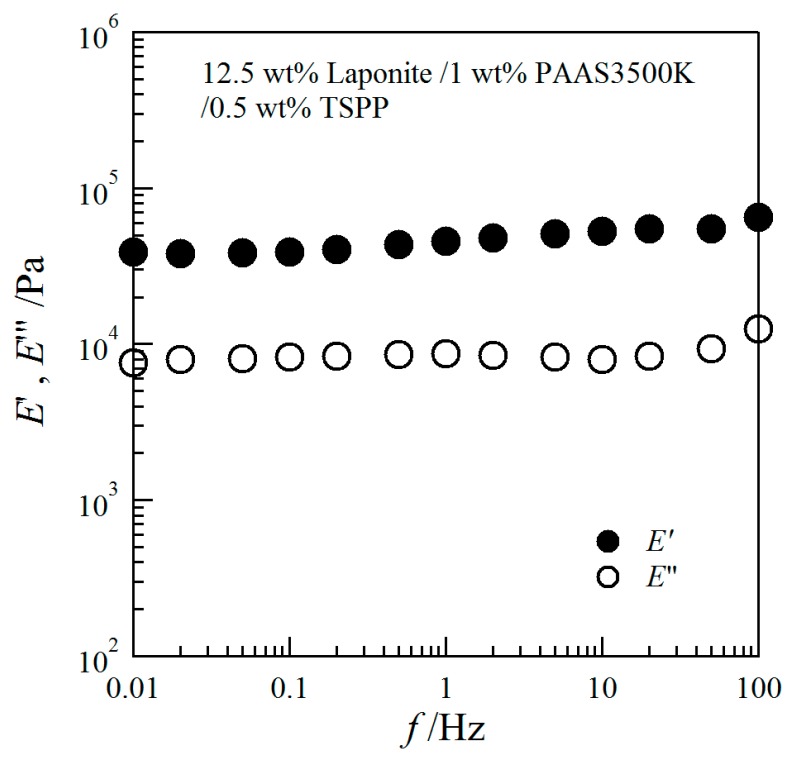
Storage modulus (*E*′) and loss modulus (*E*″) vs. frequency for a 12.5 wt % clay/PAAS3500k hydrogel.

We conducted dynamic mechanical measurements for a clay/PAAS blend hydrogel in order to ascertain whether the gel is rheologically elastic or not. In Figure 4 we show the storage modulus *E*′ and the loss modulus *E*″ for a 12.5 wt % clay/1 wt % PAAS3500K gel. The figure shows that *E*′ is larger than *E*″, and *E*′ is independent of frequencies. These results indicate that the sample is rheologically elastic.

**Figure 5:**
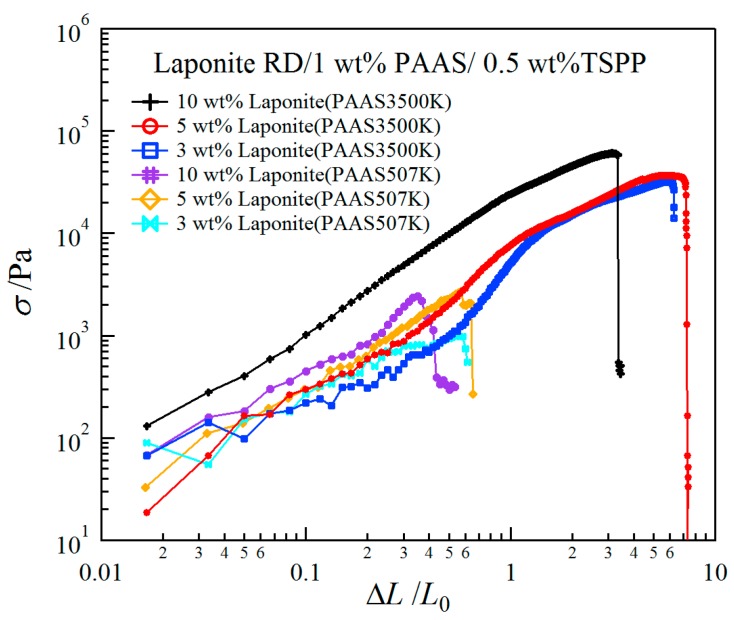
Typical tensile stress-strain curves of clay/PAAS3500K and clay/PAAS507K gels at various clay concentrations.

Figure 5 shows tensile stress-strain curves for clay/1 wt % PAAS3500K hydrogels and for clay/1 wt % PAAS507K hydrogels at various clay concentrations. The former gels were stretchable until 700% at low clay concentrations. In contrast, the latter gels with short polymer chains were fractured at short elongation. Thus the mechanical properties of the blend hydrogels composed of PAAS with molecular weights smaller than a few million grams per mole become extremely poor, as reported in a previous study [8].

The toughness of a material may be characterized by the area under the tensile stress-strain curves [15,16,17,18];

(1) $$\mathrm{toughness}=\overset{\varepsilon_{f}}{\underset{0}{\int }}\sigma d\varepsilon$$

where *ε_f_* represents the strain at break. We estimated the ratios of the toughness for clay/1 wt % PAAS3500K hydrogels to that of clay/1 wt % PAAS507K hydrogels at various clay concentrations. These values were evaluated from the average of 4–6 tensile measurements. The ratios attained 434, 206 and 548, respectively, for hydrogels with 3, 5 and 10 wt % clay concentrations; thus it is necessary to use PAAS with ultra-high molecular weight to acquire toughness for the clay/PAAS blend hydrogels.

### 2.3. Structures of Clay/PAAS Hydrogels

In order to investigate the structures of the hydrogels, we conducted synchrotron SAXS measurements for clay/PAAS3500K and clay/PAAS507K hydrogels. Since the blend hydrogel used in this study is a four-component system composed of clay, PAAS, TSPP, and water, the whole X-ray scattering intensity can be described by a sum of several partial scattering functions [19]. However, as the X-ray scattering length of clay is much larger than those of water and PAAS [6], the SAXS intensity of the blend hydrogel may be approximated by the scattering intensity from clay particles, i.e., the partial scattering function from clay-clay correlation. Thus, the SAXS intensity of the blend hydrogel *I*(*q*) can be approximately described by the scattering function from a two-component system.

(2) $$I\left(q\right)=Kn_{\mathrm{clay}}V_{\mathrm{clay}}^{2}\Delta \rho^{2}P\left(q\right)S_{\exp}\left(q\right)$$

Here *K* is an experimental constant, and *n*_clay_ denotes the number density of clay particles with the volume *V*_clay_. Δ*ρ*, *P*(*q*), and *S*_exp_(*q*) are the scattering length density difference between the clay particles and the matrix, the form factor of the clay particles, and the experimental structure factor, respectively [20,21,22,23,24]. Here the magnitude of the wavevector *q* is defined by the following equation.

*q *= 4πsin(*θ*/2)/*λ* (3)

here *θ* and *λ* are the scattering angle and wavelength of the X-rays, respectively. The clay particles used in this study have disk-like shapes with a thickness of ~10 Å and a radius of 120–150 Å [20,25]. The form factor of randomly distributed disk-shaped particles *P*(*q*) is presented in the following form [26];

(4) $$P(q)=4\int_{0}^{\pi /2}\left[\frac{\sin^{2}\left(qH\cos\beta \right)}{{\left(qH\cos\beta \right)}^{2}}\right]\left[\frac{J_{1}^{2}\left(qR\sin\beta \right)}{{\left(qR\sin\beta \right)}^{2}}\right]\sin\beta d\beta$$

where *R* and 2*H* denote the radius and the thickness of disk-shaped particles, respectively, and β is the angle between the scattering vector *q* and the axis of the disk-shaped particles. *J*_1_ represents the first-order Bessel function. In a previous study, we obtained the structural parameters of the clay particles from SAXS data of a dilute clay dispersion, where the polydispersity of the radius *R* with a Gaussian distribution was taken into consideration (2*H* = 10 Å, *R* = 130 Å, and the standard deviation in the polydispersity of the radius = 30 Å) [5].

**Figure 6:**
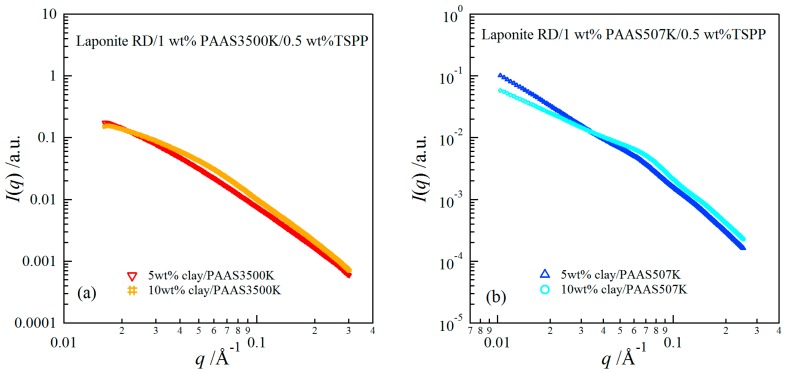
SAXS curves for clay/PAAS3500K gels (**a**) and for clay/PAAS507K gels (**b**) at 5 wt % and 10 wt % clay concentrations.

Figure 6 depicts SAXS profiles of clay/PAAS3500K and clay/PAAS507K hydrogels with 5 wt % and 10 wt % clay concentrations. Although the SAXS intensity for the hydrogel with 10 wt % clay was larger than that for hydrogel with 5 wt % clay at high *q* values, the former was weaker than that of the latter at small *q* values, where interparticle interference effects become important. This result indicates that the effects of the interparticle interference were stronger at the higher clay concentration, i.e., the interparticle interactions were more repulsive at the higher clay concentration. In this context, the SAXS data for the clay/PAAS3500K gel and for the clay/PAAS507K gel show the same trend.

**Figure 7:**
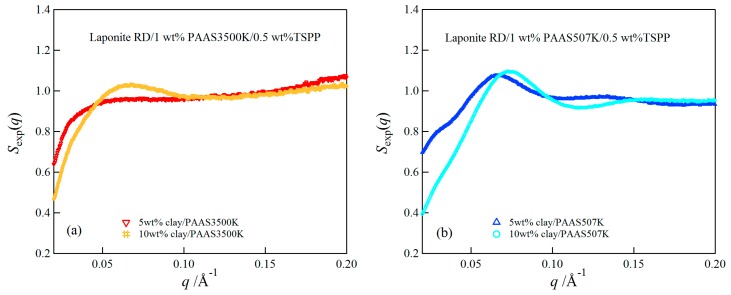
The experimental structure factors of clay/PAAS3500K gels (**a**) and clay/PAAS507K gels (**b**) at 5 wt % and 10 wt % clay.

We have estimated the experimental structure factors for both clay/PAAS3500K gel and clay/PAAS507K gel by dividing the scattering intensity by the calculated *P*(*q*). Figure 7 depicts the experimental structure factors for clay/PAAS3500K gels (a) and clay/PAAS507K gels (b) with 5 wt % and 10 wt % clay concentrations. A decrease of *S*_exp _(*q*) in the small *q* for the clay/PAAS507K gel with 10 wt % clay was greater than that of the clay/PAAS3500K gel with the same clay concentration. This result may indicate that the interparticle interactions are more repulsive for clay/PAAS507K gel than clay/PAAS3500K gel at the high clay concentration. Such repulsive interactions may be due to a steric repulsion of the polymer chain adsorbed on the edges of the clay particles or electrostatic repulsion resulting from the double layer counter ion distribution. However, it is difficult to clarify the local structure such as the adsorbed polymer layer from the SAXS data, because the SAXS intensity mainly comes from the clay-clay correlation, as mentioned above. In order to investigate the structures of the adsorbed polymer layer, it may be necessary to use a technique such as contrast variation small-angle neutron scattering [27]. The study of the local structure will be a subject of future work.

## 3. Summary

We studied the mechanical, swelling, and structural properties of clay/PAAS blend hydrogels prepared by simple blending. The swelling-deswelling behavior was observed for all the clay/PAAS blend hydrogels investigated in this study. Both the maximum and the constant values of the swelling ratios decreased with the increase of the clay concentrations and increased with the increase of the PAAS concentrations. These results were attributed to the increase of cross-linking densities with the increase of the clay concentrations and the increase of the contribution of mobile ions to the osmotic pressure with the increase of the PAAS concentrations. The toughness of the clay/PAAS blend hydrogels depends upon the molecular weight of the constituent polymer, and it has been demonstrated that the toughness of the hydrogel with an ultra-high molecular weight polymer (*M*_w_ = 3.50 × 10^6^) was several hundred times greater than that of the hydrogel with a moderate molecular weight polymer (*M*_w_ = 5.07 × 10^5^). SAXS results for both the clay/PAAS3500K and clay/PAAS507K gels showed that the interparticle interactions are more repulsive at higher clay concentrations, and the repulsive interactions was greater for the gel with the moderate molecular weight PAAS than for the gel with the ultra-high molecular weight PAAS.

## 4. Experiments

### 4.1. Samples and Sample Preparation

Laponite RD (Na_0.7_[(Si_8_Mg_5.5_Li_0.3_)O_20_(OH)_4_]) was kindly supplied by RockWood Ltd., Aomori Prefecture, Japan. PAAS with weight-average molecular weight of 3.50 × 10^6^ (from Wako Pure Chemical Industries) and 5.07 × 10^5^ (from American Polymer Standards) were used in this study, and they are hereafter denoted as PAAS3500K and PAAS507K, respectively. TSPP decahydrate as a dispersant of clay particles was obtained from Kanto Chemicals Co.

The clay/PAAS hydrogels were prepared in the manner mentioned below. After clay (Laponite RD) was added to a TSPP aqueous solution, the solution was stirred for more than 20 min. Then the clay solution was added to a PAAS solution. The final concentrations of PAAS and TSPP are 1 wt % and 0.5 wt %, respectively, unless their concentrations are not described in the text or the figure. The prepared mixture was degassed by centrifugation for 15 min at 7500 rpm. Both the samples for the tensile tests and for synchrotron SAXS experiments were placed in a spacer with a thickness of 1 mm. The samples were sandwiched between Teflon sheets, and then a weight was placed on them. After the samples were placed for 2 days at room temperature (ca. 22 °C), they were used for tensile or SAXS measurements.

### 4.2. Mechanical Measurements

Tensile tests were conducted using an ORIENTEC TENSILE TESTER STM-20 at a stretching speed of 10 mm/min. The cross-sectional area of the undeformed gel was used for the calculation of the stress. Tensile stress-strain (Δ*L*/*L*_0_) curves were measured for clay/PAAS3500K and clay/PAAS507K hydrogels, where *L*_0_ and Δ*L* (=*L* − *L*_0_) denote the initial length of the gel and the deformation, respectively.

We conducted dynamic mechanical measurements with a compression mode for a 12.5 wt % clay/PAAS3500K blend hydrogel. The storage moduli *E*′ and loss moduli *E*″ were obtained as a function of the frequency in the range of 0.01–100 Hz by using a rheometer (Seiko Instruments, DMS 6100).

### 4.3. Swelling Measurements

We investigated the time course of swelling ratios of the clay/PAAS3500K blend hydrogels at different clay concentrations and at different PAAS concentrations. Cylindrical samples with a diameter of 6 mm and a length of 8 mm were used for the swelling measurements. The samples were placed in a large amount of water without replacing water, and the swelling ratios of the gels were measured as a function of time. The swelling ratios were estimated from the ratio of the weight of the swelled gel *W*(*t*) to that of the dried gel *W*_dry_.

### 4.4. Synchrotron Small-Angle X-ray Scattering

We conducted synchrotron SAXS experiments at the beam-line 6A and 10C (BL6A and BL10C) of the Photon Factory at the High Energy Acceleration Research Organization in Tsukuba, Japan. The SAXS measurements were performed with a wavelength of 1.5 Å and with a two-dimensional detector, Pilatus 1 M. The detected two-dimensional SAXS images were circular-averaged with a software (Fit2D V12.077 by A. Hammersley [28]) for small-angle scattering data analysis, so that the scattering intensity was obtained as a function of the magnitude of wave vector *q*. The scattering profile was corrected for background scattering.
